# The Link between Oxidative Stress, Mitochondrial Dysfunction and Neuroinflammation in the Pathophysiology of Alzheimer’s Disease: Therapeutic Implications and Future Perspectives

**DOI:** 10.3390/antiox11112167

**Published:** 2022-10-31

**Authors:** Maria Carolina Jurcău, Felicia Liana Andronie-Cioara, Anamaria Jurcău, Florin Marcu, Delia Mirela Ţiț, Nicoleta Pașcalău, Delia Carmen Nistor-Cseppentö

**Affiliations:** 1 Faculty of Medicine and Pharmacy, University of Oradea, 410073 Oradea, Romania; 2 Department of Psycho-Neuroscience and Rehabilitation, Faculty of Medicine and Pharmacy, University of Oradea, 410073 Oradea, Romania; 3 Department of Pharmacy, Faculty of Medicine and Pharmacy, University of Oradea, 410028 Oradea, Romania

**Keywords:** Alzheimer’s disease, oxidative stress, mitochondrial dysfunction, neuroinflammation, antioxidants, stem cell therapies, nanotechnology

## Abstract

Alzheimer’s disease (AD), the most common form of dementia, has increasing incidence, increasing mortality rates, and poses a huge burden on healthcare. None of the currently approved drugs for the treatment of AD influence disease progression. Many clinical trials aiming at inhibiting amyloid plaque formation, increasing amyloid beta clearance, or inhibiting neurofibrillary tangle pathology yielded inconclusive results or failed. Meanwhile, research has identified many interlinked vicious cascades implicating oxidative stress, mitochondrial dysfunction, and chronic neuroinflammation, and has pointed to novel therapeutic targets such as improving mitochondrial bioenergetics and quality control, diminishing oxidative stress, or modulating the neuroinflammatory pathways. Many novel molecules tested in vitro or in animal models have proven efficient, but their translation into clinic needs further research regarding appropriate doses, delivery routes, and possible side effects. Cell-based therapies and extracellular vesicle-mediated delivery of messenger RNAs and microRNAs seem also promising strategies allowing to target specific signaling pathways, but need further research regarding the most appropriate harvesting and culture methods as well as control of the possible tumorigenic side effects. The rapidly developing area of nanotechnology could improve drug delivery and also be used in early diagnosis.

## 1. Introduction

Alzheimer’s disease (AD) is a major form of neurodegenerative disease and the most common form of dementia. It affects around 50 million persons worldwide, but the incidence is expected to triple by 2050 due to the natural aging of the population, with the greatest increase in incidence being expected in low- and middle-income countries [1]. Currently, the prevalence in European countries is estimated to be around 4.4% in people over 65, while in the USA the prevalence already reached 9.7% in persons over 70 years of age [2,3]. Because almost half of AD patients require a high level of care, the disease poses heavy economic burden on both society and family [4]. The mortality rates also steadily increase, making AD the sixth cause of mortality in the United States of America (USA) [5].

The clinical hallmarks of AD are memory deficits and executive dysfunctions [6], while pathology is characterized by deposition of extracellular beta-amyloid in the form of senile plaques and intracellular accumulation of neurofibrillary tangles [7], noted already by Alois Alzheimer in the original description of the disease [8].

Much research has focused on elucidating the complex molecular mechanisms leading to the development of the disease. For years the scientific community has been divided into two camps: those who considered tau hyperphosphorylation and aggregation of the misfolded protein to be the primary culprit (“Tauists”) [9] and those who viewed Aβ accumulation in senile plaques as the central anomaly (“Baptists”) [10]. More recently, oxidative stress has been increasingly implicated in the pathogenesis of many neurodegenerative diseases, AD included [11]. Although augmented in the presence of fibrillary amyloid beta (Aβ), oxidative stress appears to precede the deposition of Aβ [12], which could be one of the possible reasons for the repeated failures of anti-amyloid therapeutic strategies.

The present article reviews the pathogenic cascades involving mitochondrial dysfunction and oxidative stress in AD pathogenesis and their connection to the chronic inflammatory state of the central nervous system and highlights novel therapeutic targets and strategies which could be employed in the attempt to offer disease-modifying agents for the treatment of this devastating neurodegenerative disease.

## 2. Oxidative Stress

Reactive oxygen species (ROS), such as superoxide (O_2_**^•−^**), hydroxyl radicals (HO**^•^**), peroxyl radicals (RO_2_^•^), hydrogen peroxide (H_2_O_2_), organic peroxides (ROOH), or peroxynitrite (ONOO^−^), as well as reactive nitrogen species (RNS), which include nitric oxide (NO**^•^**), nitrogen dioxide (NO_2_**^•^**), nitrous acid (HNO_2_), or, again, peroxynitrite, are continuously generated in living organisms and serve as signaling molecules [13]. Aside from their short half-life, their action is rapidly terminated by potent antioxidant defense systems which inactivate these molecules. Oxidative stress ensues in conditions of excessive ROS or RNS generation or when the antioxidant defenses are deficient, leading to damage inflicted upon a wide range of biomolecules [14].

ROS emerge from many sources in the cerebral parenchyma. The main source appears to be the mitochondrial activity, mainly through complex I (NADH dehydrogenase) and III (ubiquinone cytochrome c reductase) of the electron transport chain (ETC) [15]. However, other mitochondrial enzymes also generate significant amounts of ROS, such as monoamine oxidase, glycerol phosphate dehydrogenase, α-ketoglutarate dehydrogenase, and p66shc [16]. Various metabolic factors, such as the NADH/NAD^+^ ratio, mitochondrial calcium concentration, or the mitochondrial membrane potential (∆ψm) influence the rate of ROS production [11]. ROS may also stem from the activity of NADPH oxidase (NOX), the monoamine oxidases (MAO A and B) or several peroxisomal enzymes, such as xanthine oxidase, D-aspartate oxidase, acyl CoA oxidases, D-amino acid oxidase, urate oxidase, or L-α-hydroxy oxidase [11].

The particular vulnerability of the brain to ROS-induced damage is due to a series of particularities [17,18]:

- The brain has high energy demands required to maintain the ionic gradients and support synaptic transmission [19].
- Low antioxidant defenses (neurons have 50 times less catalase than hepatocytes and 50% lower cytosolic glutathione than other cells) [20].
- The metabolism or auto-oxidation of neurotransmitters, such as dopamine, serotonin or adrenaline can also generate ROS [17].
- The brain is enriched in transition metals, such as Cu^+^ and Fe^2+^, which act as catalyzers in the hydroxyl-generating Fenton reaction [21].
- Brain cells have a high membrane surface/cytoplasmic volume ratio and cell membranes are rich in cholesterol, which may undergo auto-oxidation [22], as well as polyunsaturated fatty acids, which are very susceptible to free radical-induced peroxidation.

Assessment of oxidative stress relies on identification and measurement of the molecules resulted from oxidation of these various targets. Peroxynitrite (resulting from the reaction of superoxide and nitric oxide) attacks proteins especially at tyrosine residues and generate 3-nitrotyrosine, while protein carbonyls result from free radical-induced scission of the peptide chains. Lipid peroxidation (abstraction of allylic hydrogen atoms from side chains of phospholipids by ROS and RNS) leads to the generation of lipid peroxides. Isoprostanes, derived from arachidonic acid, eicosapentaenoic or docosahexaenoic acid are regarded as the most reliable biomarkers of lipid peroxidation. ROS also attack nuclei acids. Oxidative DNA damage of guanine residues results in 8-hydroxy-deoxyguanosine (8OHdG), while 8-hydroxyguanine (8OHG) indicates ROS and RNS attack on guanine residues in RNA [13]. Commonly used biomarkers for carbohydrate oxidative damage are advanced glycation end products (AGEs) [23]. Table 1. provides an overview of the oxidative alterations of various biomolecules and the commonly used derivatives for quantification of oxidative stress-induced damage.

## 3. Oxidative Stress and Mitochondrial Dysfunction in Alzheimer’s Disease

Compelling evidence implicates oxidative stress in the pathophysiology of AD, promoting tau hyperphosphorylation and neurofibrillary pathology by inhibition of phosphatase 2A, which activates glycogen synthase kinase 3β (GSK3β) [24,25], and accumulation of Aβ by ROS-induced inhibition of the proteasomal system through impaired mammalian target of rapamycin (mTOR) signaling [26]. Increased levels of malondialdehyde and 4-hydroxy-2-nonenal have been detected in brain tissue and cerebrospinal fluid (CSF) samples of AD patients [27,28], together with high plasma levels of AGEs and protein carbonyls in male patients [29], while the levels of antioxidant enzymes, such as glutathione peroxidase, catalase, or superoxide dismutase were reduced in areas of the brain affected by AD pathology [30].

Although oxidative stress has many sources and interferes with many pathways, to keep the present review from being extensively exhaustive, we will focus on the complex relationships between oxidative stress, mitochondrial dysfunction, cellular protein homeostasis and neuroinflammation.

### 3.1. Oxidative Stress and Mitochondrial Dysfunction in Alzheimer’s Disease

#### 3.1.1. Oxidative Stress and Normal Aging

Aside from the small percentage of early onset AD, in which patients show specific genetic anomalies, up to 80% of cases occur in people aged 75 years or older [31], which makes aging one of the most prominent risk factors.

Aging occurs at molecular and cellular levels in the brain [32]. The hypothalamus, through the projections of the orexinergic nucleus to the reticular activating system, the secretion of neurohormones, and the connections with the endocrine system, initiates the gradual decline of the energy metabolism of the entire body, brain included [33,34]. Additional factors, such as decreased oxygen supply due to changes in the cerebral vasculature, may further accelerate the decline in metabolism [11].

Neurons, being among cells with highest energy demands to fuel synaptic transmission and neural plasticity [35], rely mainly on mitochondria and oxidative phosphorylation (OXPHOS) to supply the necessary amounts of adenosine triphosphate (ATP) and have more mitochondria than most other cells, distributed mainly at synapses [36]. With aging, mitochondria exhibit a series of morphological changes, such as fragmentation of cristae and disappearance of the inner membrane vesicles. At a molecular level, dissociation of ATP synthase dimers alters the capacity of the organelles to maintain normal energy supply [36]. Furthermore, aging associates an impaired mitochondrial homeostasis, with predominance of fission over fusion, thereby preventing the repair of damaged mitochondria [37]. In addition, age-dependent build-up of oxidative damage in mitochondrial DNA (mtDNA), more susceptible to ROS attack due to the lack of protective histones [38], and an impairment of the repair mechanisms through defective proteins (ex: Ku86) and altered activity of RNA polymerase II [39], results in altered expression of mitochondrial-encoded OXPHOS enzymes, further reducing the rate of ATP production. Oxidative damage also builds up on nuclear pores, causing loss of nuclear pore proteins, such as Nup93, and altering nucleocytoplasmic transport [40]. Aging associates a significant decline in nicotinamide adenine dinucleotide (NAD+), an essential co-factor of sirtuins (a group of histone deacetylases that upregulate antioxidant enzymes such as catalase and manganese superoxide dismutase), possibly due to consumption by activated poly(ADP-ribosyl) polymerases (PARPs) [41]. The involvement of oxidative stress in aging is demonstrated once again by the effect of klotho, a transmembrane protein hormone originally recognized as an aging suppressor. Subsequently, it was shown that klotho regulates intracellular signaling pathways that regulate the oxidative stress response, inflammation, DNA damage and various types of cell death [42].

#### 3.1.2. Oxidative Stress and Energetic Failure in Alzheimer’s Disease

Imaging studies performed in vivo using fluorodeoxyglucose PET convincingly demonstrate regional low glucose consumption [43], presumably due to reduced glycolysis, neuronal loss, and synaptic dysfunction [44]. Although the primary insult leading to AD is still a matter of debate, these metabolic changes indicate a crucial role for mitochondrial dysfunction in AD pathogenesis, leading some researchers to postulate the “mitochondrial cascade hypothesis” [45].

With aging, cerebral glucose metabolism progressively declines as a result of changes in brain insulin and cortisol levels [46] and of decreased activity of synaptic ATPases [47], leading to a 45% reduced cerebral glucose utilization [46]. In late stages of AD, the reduction is even more pronounced, reaching about 55% [48].

A series of enzymes of the ETC and Krebs cycle, such as cytochrome c oxidase (complex IV), ATP synthase (complex V), pyruvate dehydrogenase, phosphoglycerate kinase, phosphoglycerate mutase, or α-ketoglutarate dehydrogenase are dysfunctional, presumably as a result of oxidation and nitration, contributing to impaired energy production [44]. Subcortical cerebral regions of AD patients show increased activity of the glycolysis-initiating enzyme hexokinase, as well as of lactate dehydrogenase, the latter suggesting a switch to anaerobic respiration to compensate for impaired ATP generation via aerobic metabolism [46]. It is still under debate whether Aβ and tau play a direct or indirect role in the reduction of the activity of these enzymes. However, by incubating cerebral mitochondria from rat brains with Aβ25-35 and Aβ1-42 with or without nitric oxide, Casley and colleagues demonstrated that both Aβ peptides inhibited mitochondrial respiration, but addition of nitric oxide substantially augmented this inhibition [49].

Considering that mitochondrial ETC complexes I and IV are among the main sources of ROS [50] and taking into account the very short half-life of ROS and their physical proximity with mitochondrial proteins, it is reasonable to assume that these proteins are first targets of oxidative attack. In advanced stages of AD, brain samples from patients show significant nitration and lipoxidation of ATP synthase, as well as oxidative alterations of aldolase, glyceraldehyde-3-phosphate dehydrogenase (GAPDH), α-enolase, and phosphoglycerate mutase 1 (PGAM1) [13,51,52]. After intracellular accumulation of amyloid precursor protein (APP) and Aß, soluble Aß oligomers and APP associate to the membrane import channels blocking the entry and function of the ETC enzymes, further augmenting the generation of ROS in a vicious cycle [53]. The increased ROS levels lead to membrane lipid peroxidation, intracellular protein and nucleic acid oxidation [54], as well as APP proteolysis and Aβ generation [55].

Although mtDNA lesions can be inherited, and asymptomatic adult offspring of female AD patients exhibit reduced cytochrome c oxidase activity [56], the ROS-induced changes of these mitochondrial enzymes cannot be overlooked. MtDNA is a circular double-stranded DNA that possesses 37 genes, 13 of which encode for components of the ETC [36]. Lacking protective histones, and having less efficient DNA repair mechanisms, mtDNA is ten times more prone to mutations than nuclear DNA. These mutations can further propagate through clonal expansion and exacerbate mitochondrial dysfunction culminating in cell death [36].

The reduced levels of ATP impair synaptic plasticity at glutamatergic synapses leading to impaired long-term potentiation and long-term depression, manifested clinically as learning and memory deficits. Together with oxidative changes of mitochondrial proteins, the ETC dysfunctions alter the ∆ψm, causing opening of the mitochondrial permeability transition pore (MPTP) and release of apoptosis-inducing factors which will lead to neuronal loss [57].

#### 3.1.3. Oxidative Stress and Impaired Mitochondrial Quality Control

In order to meet energy demands, the size, shape, and number of mitochondria must be tightly regulated. Mitochondrial biogenesis is controlled mainly by peroxisome proliferator-activated receptor gamma coactivator 1α (PGC-1α), a transcriptional coactivator which together with different transcription factors (such as nuclear respiratory factor 1—Nrf1 and nuclear respiratory factor 2—Nrf2), coordinates the expression of nuclear-encoded mitochondrial genes, as well as of mitochondrial transcription factor A (TFAM), a transcription factor that regulates mtDNA transcription [58]. PGC-1α activity is influenced by ATP demand, intracellular calcium concentrations, various cytokines, and ROS, among others [59]. Fusion and fission are two opposite processes which also contribute to mitochondrial quality control. Through fusion, two mitochondria unite, sharing essential components. The process is regulated by two GTPases, Mfn1 and Mfn2 (mitofusins 1 and 2), which control fusion of the outer mitochondrial membrane (OMM), and another GTPase, OPA1 (optic atrophy 1), which regulates fusion of the inner mitochondrial membrane (IMM) [60]. Fission, the process through which mitochondria are spliced, is regulated by two proteins: Drp1 (dynamin-related/-like protein 1) and Dnm2 (dynamin 2) and a series of adaptor proteins such as MFF (mitochondrial fission factor), Fis1 (mitochondrial fission protein 1) and mitochondrial dynamics proteins 49 and 51 (MiD49 and MiD51) [61]. Finally, irreversibly damaged mitochondria are disposed through mitophagy, in which a number of proteins are sequentially recruited and activated, leading to the formation of an isolation membrane and fusion of the autophagosome with lysosomes [62].

PGC-1α is abundantly expressed in tissues with high energy demand, but both AD patients and transgenic mouse AD models exhibited reduced expression of PGC-1α [63]. Interestingly, PGC-1α is increased by SIRT1 activation through caloric restriction, while decreased PGC-1α levels are associated with impaired brain insulin signaling, observations which could explain obesity and diabetes mellitus being risk factors for AD [64]. Further, most of the mitochondrial proteins are encoded by nuclear DNA and must be imported, making mitochondrial biogenesis heavily dependent on the protein import machinery [65]. The main entry gate is the translocase of the outer membrane (TOM), which consists of a pore-forming protein TOM44 and three receptor proteins on the cytosolic side (TOM20, TOM22, and TOM70) [65]. In AD there is a reduced expression of TOM22 and TOM70 [66], and oxidative stress augments the inhibition of the protein import system [67]. In addition, APP forms stable complexes with translocases of the OMM and IMM while Aβ translocates to mitochondria and localizes to the cristae, further impairing the import of essential mitochondrial proteins [65].

The balance between mitochondrial fusion and fission is crucial for maintaining a healthy pool of mitochondria with proper distribution. Early studies revealed altered size and number of mitochondria in susceptible pyramidal neurons in brain biopsy samples of AD patients, indicative of a fragmented mitochondrial network [68]. Moreover, a peculiar mitochondrial shape, termed “mitochondria-on-a-string”, was described in brain tissue from AD patients and mouse models of AD, consisting of teardrop shaped mitochondria (0.5 μm in diameter) connected by a thin double membrane extending up to 5 μm in length, and considered to be a phenotype associated with fission arrest [8,69]. Biochemical analyses revealed reduced expression of OPA1 and mitofusins, and increased levels of Drp1 andFis1 in AD brains [70]. Drp1 and Mfn2 are substrates for calpain, an enzyme that has been found activated in AD, suggesting that calpain-mediated cleavage could be, at least partly, responsible for the reduced levels of these GTPases [71]. In addition, Aβ can induce S-nitrosylation of Drp1 and lead to its increased translocation to mitochondria [72]. In later stages of AD, Drp1 forms complexes with hyperphosphorylated tau which exacerbate Aβ-Drp1 interactions, leading to increased mitochondrial fission [73]. Calcium signaling and oxidative stress are important contributors to Aβ-induced mitochondrial fragmentation. Aβ induces mitochondrial calcium influx and calcium/calmodulin-dependent protein kinase II (CAMKII)-mediated protein kinase B (Akt) activation, thereby causing Drp1 phosphorylation and increasing its mitochondrial translocation [74]. Increased oxidative stress activates extracellular signal-regulated kinase (ERK), which also results in Drp1 phosphorylation and mitochondrial translocation [75].

Normal mitochondrial fusion requires maintenance of the ∆ψm for post-translational OPA1 processing [76]. Aside from the reduced expression of proteins regulating the fusion process, overexpression of tau protein and hyperphosphorylation of tau have additional contributions to the generation of the fragmented mitochondrial network [77]. By stabilizing the actin cytoskeleton, tau disrupts the physical association of mitochondria and Drp1, preventing excessive fission. Hyperphosphorylated tau has reduced affinity for the microtubule network, promoting fission. Indeed, genetic tau ablation in mice led to increased mitochondrial fusion, decreased fission, decreased ROS production and enhanced ATP generation [78].

**Figure 1:**
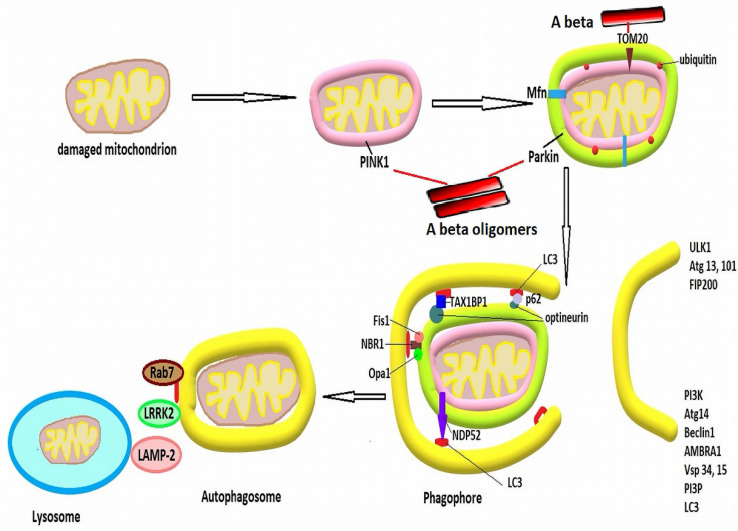
PINK1 accumulates on the OMM of damaged mitochondria, followed by recruitment and phosphorylation of Parkin which ubiquitinates OMM proteins such as the translocase of the outer mitochondrial membrane 20 (TOM20) and mitofusins (Mfn1 and Mfn2). The pre-initiation complex containing ULK1 (Unc 51-like kinase 1), Atg (autophagy related proteins) 13 and 101 and FIP200 (focal adhesion kinase family interacting partner 200) is activated, followed by recruitment of phosphatidyl inositide 3 kinase (PI3K), Atg14, beclin 1, AMBRA1 (autophagy and beclin 1 regulator 1), vascular sorting proteins Vsp34 and 15, and LC3 (light chain 3) leading to generation of phosphatidyl inositol-3-phosphate (PI3P). The ubiquitinated OMM proteins recruit autophagy adaptor proteins (NRB1—neighbor of BRCA1 gene 1 protein, optineurin, TAX1BP1—Tax 1 binding protein 1, NDP52—nuclear dot protein 52, p62), which interact with LC3 leading to closure of the of the phagophore and formation of the autophagosome. Rab7, a lysosome-associated small GTPase, LRRK2 (leucine rich repeat kinase 2) and LAMP2 (lysosome-associated membrane protein 2) mediate fusion of the autophagosomes with lysosomes. In AD, Aβ can be internalized by mitochondria via TOM, damaging the organelle, while cytoplasmic Aβ decreases the levels of PINK1 and parkin, thereby impairing the mitophagy pathway.

Irreversibly damaged mitochondria are disposed through a specific form of autophagy, termed mitophagy. The most well described, non-receptor-mediated mitophagy pathway begins with PTEN-induced kinase 1 (PINK1), which accumulates on the OMM, facilitated by the impaired ∆ψm. PINK1 recruits and phosphorylates Parkin, which ubiquitinates OMM proteins, such as voltage-dependent anion channels 1 (VDAC1), Mfn1 and Mfn2 as well as TOM20, blocking the fusion process and allowing for isolation of the mitochondria [11]. The ubiquitinated proteins recruit autophagy adaptor proteins, such as OPTN (optineurin), NDP52 (nuclear dot protein 52), NBR1 (neighbor BRCA1), sequestosome-1, or TAX1BP1 (Tax-1 binding protein), which in turn interact with autophagosome proteins like GABARAP (γ-aminobutiric acid type A-receptor-associated protein) or LC3 (light chain 3) through LC3 interacting regions to mediate autophagosome formation and fusion with lysosomes. In the Parkin-independent mitophagy pathway, a series of proteins of the OMM, such as AMBRA1 (autophagy, and beclin 1 regulator), FUNDC1 (FUN14 domain-containing protein 1), NIX (Nip3-like protein), and BNIP3 (Bcl2 interacting protein 3), or of the IMM, such as cardiolipin and prohibitin 2 (PHB2), through phosphorylation or dephosphorylation increase their affinity and bind to LC3 and subsequently mediate fusion of mitochondria with lysosomes [62,79]. Figure 1. provides a schematic overview of the mitophagy process.

Another method of mitochondrial disposal is transcellular mitophagy, a process through which neurons extrude damaged mitochondria which will be endocytosed by glial cells, transferring this energetically demanding task to other cells [62]. In turn, glial cells can transfer healthy mitochondria to neurons, protecting the latter from energetic failure [80]. Research suggests a significant failure of mitophagy in AD, swollen mitochondria with distorted cristae being identified in biopsy samples of human AD cases and in transgenic animal models [81]. Increased PINK1, Parkin and ubiquitinated mitochondrial proteins were found in APP transgenic mice and in pyramidal hippocampal neurons isolated from AD patients [82], but accumulation of tau protein might increase ∆ψm, preventing PINK1 and Parkin recruitment to the OMM [83] and is also able to sequester Parkin in the cytosol via interaction with the projection domain of tau [84]. In addition, mature lysosomes are concentrated in the neuronal cell body whereas mitochondria extend along the axons and dendrites of neurons, a particularity which makes neuronal mitophagy a slower process [85].

#### 3.1.4. Oxidative Stress and Mitochondrial Trafficking

In order to meet the local energy demands mitochondria must also be trafficked along neuronal outgrowths to distant areas. The axonal transport is two-directional. Mitochondria with high ∆ψm predominantly travel in an anterograde direction, while damaged mitochondria with low ∆ψm are moved in a retrograde direction, towards the cell soma, presumably for destruction or repair [86]. The major motor for anterograde transport of mitochondria in neurons is kinesin-1, the heavy chain of which interacts with Miro, an atypical Rho GTPase located on the OMM. Miro also interacts with Milton, which in turn binds to the C-terminus of kinesin-1 heavy chain. Mitochondrial retrograde movement is driven by dynein, which forms a complex with dynactin and interacts also with Miro and Milton [87]. Additionally, anchor proteins serve for mitochondrial docking. PINK1 and Parkin phosphorylate Miro, leading to a detachment of the adaptor protein kinesin from mitochondria and arrest of the organelle [88]. Calcium signals, ROS, oxygen and ATP levels regulate Miro and Milton, determining mitochondrial movement and position. Calcium elevation can directly impact Miro, or influence downstream signaling molecules, such as GSK3β or calcineurin [89].

Besides morphological abnormalities, mitochondrial distribution is also significantly altered in AD, being less abundant in the neuronal processes of vulnerable neurons [90]. Mutations in presenilin 1, through activation of GSK3β lead to phosphorylation of kinesin light chain and release of kinesin from cargo [91]. Aβ reduces the expression of kinesin motor proteins [92] and interacts with dynein intermediate chain impairing its normal function [93]. Overexpression and phosphorylation of tau impairs mainly anterograde axonal transport of mitochondria and other vesicles through their enhanced microtubule binding [94]. The c-Jun N-terminal kinase-interacting protein 1 (JIP1), which associates with the kinesin motor protein complex [84], has also a significant contribution to mitochondrial trafficking. Abnormal hyperphosphorylated tau, aside from causing disassembly of the microtubule tracks, sequesters JIP1 in the cell body disturbing the formation of the kinesin motor complex and impacting anterograde axonal transport [95].

#### 3.1.5. Oxidative Stress and Disruption of Calcium Homeostasis

Aside from being the energy source to support membrane ion exchanges, synaptic transmission and plasticity or gene expression, healthy mitochondria also act as a high-capacity calcium buffer, maintaining cellular Ca^2+^ homeostasis [23]. Mitochondrial Ca^2+^ uptake occurs through voltage-dependent anion-selective channel proteins (VDACs), which mediate Ca^2+^ transfer into the intermembrane space, and through the mitochondrial Ca^2+^ uniporter (MCU) located on the IMM, which enables Ca^2+^ to move to the mitochondrial matrix. Increases in mitochondrial calcium augment ATP production by activating the ETC dehydrogenases [96], but Ca^2+^ overload leads to the opening of the mitochondrial permeability transition pore (MPTP), which causes mitochondrial depolarization and triggers apoptosis through the release of cytochrome c and other pro-apoptotic factors [88,97]. Calcium efflux occurs via the mitochondrial Na^+^/Ca^2+^ exchanger located in the IMM, and termed NCLX because it is also able to exchange Li^+^ for Ca^2+^, according to the electrochemical gradient of Na^+^ [98], and from the intermembrane space through the Na^+^/Ca^2+^ exchanger 3 and VDACs [99]. In maintaining Ca^2+^ homeostasis, mitochondria interact with the ER through mitochondria-associated ER membranes (MAMs), microdomains where the OMM is just 10–100 nanometers apart from the ER [100]. These areas are enriched in inositol 1,4,5-triphosphate receptors (IP3Rs) which form functional complexes with VDACs through Grp75 (glucose-regulated protein 75), a chaperone belonging to the heat shock protein 70 family [101]. IP3R-Grp75-VDAC complexes regulate Ca^2+^ transfer between the ER and mitochondria [100]. The apposition of ER to mitochondria is controlled by phosphofurin acidic cluster sorting protein 2 (PACS2) [11]. Ryanodine receptors (RyRs), also expressed by the ER, are activated by low Ca^2+^ concentrations and induce calcium-induced calcium release [18], but are inactivated by high Ca^2+^ levels, thereby preventing total depletion of the sarcoplasmic reticulum. The same receptors can sense Ca^2+^ concentrations inside the ER and release Ca^2+^ in a process known as store overload-induced calcium release. Both presenilin 1 (PSEN1) and presenilin 2 (PSEN2) localize at MAMs [102] and modulate Ca^2+^ uptake into the ER and mitochondria, with PSEN2 overexpression leading to increased mitochondrial calcium buffering, activation of calpains, and excess generation of free radicals [103]. Presenilins also interact with RyRs and increase Ca2+ release from the ER [104]. Aβ aggregates can form calcium-permeable channels in membranes [105] and can mediate Ca^2+^ transfer from ER to the mitochondria through the MCU [106], while tau inhibits mitochondrial calcium efflux [107], augmenting mitochondrial dysfunction and oxidative stress. Excessive cytosolic Ca^2+^, being a potent enzymatic activator, causes tau hyperphosphorylation, followed by tau misfolding and detachment from microtubules, translocation to the somatodendritic compartment and aggregation, resulting in neurofibrillary tangle pathology [36], thereby disrupting mitochondrial transport and increasing energy deprivation and oxidative stress [108].

#### 3.1.6. Oxidative Stress and Protein Homeostasis

For proper functioning, cells need to maintain a finely controlled balance between protein synthesis and degradation, the latter being achieved via the 20S and 26S proteasomes, the mitochondrial Lon protease, and the immunoproteasome [109].

The 20S proteasome breaks down oxidatively damaged proteins through an ATP-independent mechanism. It comprises two external rings composed of seven α subunits, and 2 core rings made up of seven β subunits, three of them exhibiting proteolytic activity. The 26S proteasome is formed through ATP-dependent addition of a 19S regulatory subunit to each of the α rings of the 20S proteasome [110]. Proteins that need to be degraded are first ubiquitinated in a step-wise manner by three enzymes—the ubiquitin-activating enzyme E1, the ubiquitin-conjugating enzyme E2, and the ubiquitin-ligase E3, after which the 19S regulatory subunits of the 26S proteasome remove the polyubiquitin chains, unfold the tagged proteins, and feed them into the catalytic core for proteolysis [111]. Increased expression of the 20S proteasome occurs mainly through activation of Nrf2. Under normal conditions, Nrf2 is bound to Keap1 (Kelch-like ECH-associated protein 1), which inhibits Nrf2 translocation to the nucleus and targets it for ubiquitinylation and proteasomal degradation [112]. During oxidative stress, Keap1 is phosphorylated and dissociates from Nrf2. Subsequently, Nrf2 is phosphorylated by Akt and migrates to the nucleus, where it binds to antioxidant response elements (AREs) within target genes and increases their expression [113]. Oxidant-induced dissociation of the 26S proteasome further increases the Nrf2 pool by preventing Nrf2 degradation [114].

Intracellular organelles do not have proteasomes and rely on proteases for damaged protein degradation. Mitochondria have many proteases but one of the best investigated is the ATP-dependent Lon P1 protease [115] which has seven monomeric subunits. Each subunit has an N-domain which interacts with the hydrophobic regions of the substrate, a serine proteolytic domain and an ATPase domain [115]. Lon P1 degrades mildly oxidized proteins, among which is aconitase, a key enzyme of the Krebs cycle [116].

After sorting in the endoplasmic reticulum and Golgi apparatus, APP is transported to synaptic terminals and is inserted into the plasma membrane as a large transmembrane protein with an extracellular and an intracellular domain [117]. APP can be cleaved by α-secretases, members of the ADAM (a disintegrin and metalloproteinase) family of proteases, within the Aβ sequence to generate soluble APPα fragments which remain in the extracellular space, modulating neuronal excitability, response to oxidative or metabolic stress, and improving synaptic plasticity, as well as a 83-amino acid carboxy-terminal fragment (αCTF) which anchors to the neurolemma and is further processed by the γ-secretase complex into an extracellular p3 fragment and an intracellular C-terminal fragment [117]. Alternatively, APP can be cleaved by BACE-1 (β-site APP cleaving enzyme) to generate the sAPPβ ectodomain and a 99 amino-acid C-terminal membrane-bound fragment that is further processed by the γ-secretase complex to generate Aβ peptides (most commonly Aβ40 and Aβ42) and the intracellular APP domain [117]. Although Aβ40 peptides tend to remain for longer in the monomer form while Aβ42 appear usually in a mix of mono-, di-, and trimers, all fragments show the tendency to oligomerize followed by the formation of fibrils and leading to the generation of amyloid plaques Aβ oligomers, aside from inducing a significant increase of ubiquitin-protein conjugates in neurons, compete against other proteasomal substrates leading to proteasomal malfunction [118]. In addition, by accumulating on mitochondrial membranes and blocking the import of mitochondrial proteins, Aβ leads to a decline in the activity of OXPHOS enzymes, weakening the mitochondrial transmembrane electrochemical gradient, thereby further diminishing the production of ATP and increasing oxidative stress and the pool of damaged proteins [119]. Due to the presence of iron–sulfur clusters at its active site, aconitase is particularly vulnerable to oxidation. If mild oxidation of the enzyme allows for rapid recognition and degradation by Lon P1, in AD aconitase is severely oxidized, which turns it into a poor substrate for Lon protease [120].

The activity of tau is regulated by its phosphorylation state, with cyclin-dependent kinase 5 and GSK3β promoting tau phosphorylation and phosphatases 2A and 2B removing the phosphate moieties [8]. Hyperphosphorylated tau, as occurs in AD, detaches from microtubules and forms oligomers and aggregates, which additionally inhibit the proteasomal activity [121].

The ubiquitin proteasome system (UPS) also influences signaling pathways that modulate neurotransmitter release and synaptic plasticity [122] such as the cAMP-dependent protein kinase A (PKA)-cAMP response element binding protein (CREB) pathway. By controlling the degradation of the PKA regulatory subunit, the UPS modulates CREB signaling, essential for memory formation and which has been shown to be impaired in AD [123].

A link between the 2 accumulated proteins in AD may be the stress-inducible regulator of calcineurin gene *RCAN1* [124]. *RCAN1* expression results in synthesis of a series of proteins that inhibit calcineurin, a serine/threonine phosphatase that dephosphorylates tau [125]. Carriers of the apoE ε4 allele, a prominent risk factor for AD, also express higher levels of *RCAN1* and, accordingly, have higher levels of phosphorylated tau [124]. Furthermore, *RCAN1* expression activates GSK3β, which induces tau phosphorylation [126]. Aβ_42_ peptide can increase the transcription of RCAN1 [127] and activates various kinases such as MAP-kinase or GSK3β [128,129], thereby accelerating tau hyperphosphorylation.

#### 3.1.7. Oxidative Stress, Transcriptional Dysregulation, and Impaired Signaling

In addition to Aβ and hyperphosphorylated tau increasing oxidative stress which further promotes Aβ accumulation and tau hyperphosphorylation in a vicious cascade, the transcription of a series of genes is dysregulated in AD, contributing to the progression of the disease. Overall, 97 genes were found to be dysregulated and associated with the clinical outcome in AD [130]. Among these impaired transcription pathways, the Nrf2/ARE pathway stands out in neurodegenerative diseases, because it regulates redox homeostasis, autophagy, mitochondrial function, and DNA repair [131].

Nrf2 is a protein of the basic leucine zipper transcription factors that forms heterodimers with small musculoaponeurotic fibrosarcoma proteins (sMAF). Subsequently, the Nrf2/sMAF heterodimers regulate the transcription of proteins that favor cell survival by binding to ARE. Nrf2 has a short half-life in the cytoplasm (less than 20 min), being sequestered by Keap1 which promotes Nrf2 ubiquitination and proteasomal degradation [132]. Oxidative modification of cysteines in certain domains of Keap1 changes its conformation favoring Nrf2 dissociation. However, Keap1-independent pathways for Nrf2 activation have also been described [131]. Phosphorylation of Nrf2 by protein kinase C (PKC), casein- kinase 2, or MAPK activates the protein and enhances its nuclear translocation [133,134]. After release from Keap1 and activation, Nrf2 associates with co-activators such as MAFs or CBP (CREB binding protein)/p300 and chromatin remodelers, followed by binding to ARE sites where it initiates the transcription of a series of crucial genes for cell survival and lead to synthesis of SOD, catalase, glutathione S-transferase, glutathione, glutathione reductase, thioredoxins and peroxiredoxins, heme oxygenases (HO), or NADPH-regenerating enzymes [131,135]. Interestingly, Nrf2 and NF-κB compete for binding CBP/p300, explaining the suppression of the Nrf2/ARE pathway in cases of severe inflammation [136].

In AD, the failure of the Nrf2/ARE signaling pathway leads to a switch of the enzymatic function of several kinases and promotes amyloidogenic cleavage of APP as well as tau phosphorylation [131,137]. Immunochemistry studies of post-mortem tissue samples from AD patients compared to age-matched controls revealed that Nrf2 is localized mainly in the cytoplasm and is not translocated to the nucleus in hippocampal neurons and astrocytes [138]. Furthermore, overexpression of APP and Aβ downregulates the Nrf2 levels [139] and impairs the expression of rescue genes, an effect which together with impaired transcription of subunits of the ETC leads to mitochondrial dysfunction and additionally increases oxidative stress [140].

Another impaired signaling pathway in AD is the tyrosine kinase B (TrkB) pathway, closely connected to brain-derived neurotrophic factor (BDNF). By binding BDNF, TrkB activates the PI3K/Akt pathway, the MAPK pathway, and the phospholipase C-γ pathway through which BDNF promotes neuronal survival and growth as well as synaptic plasticity [141]. The TrkB and Nrf2/ARE pathways are closely connected, with the TrkB pathway being probably upstream of the Nrf2/ARE pathway because Akt phosphorylation mediates the nuclear translocation of Nrf2 [142]. Despite conflicting reports, it appears that BDNF is downregulated in the cortex, hippocampus, cerebrospinal fluid (CSF) and blood of AD patients [143]. Both Aβ and tau protein contribute to decreased BDNF transcription [144,145].

**Figure 2:**
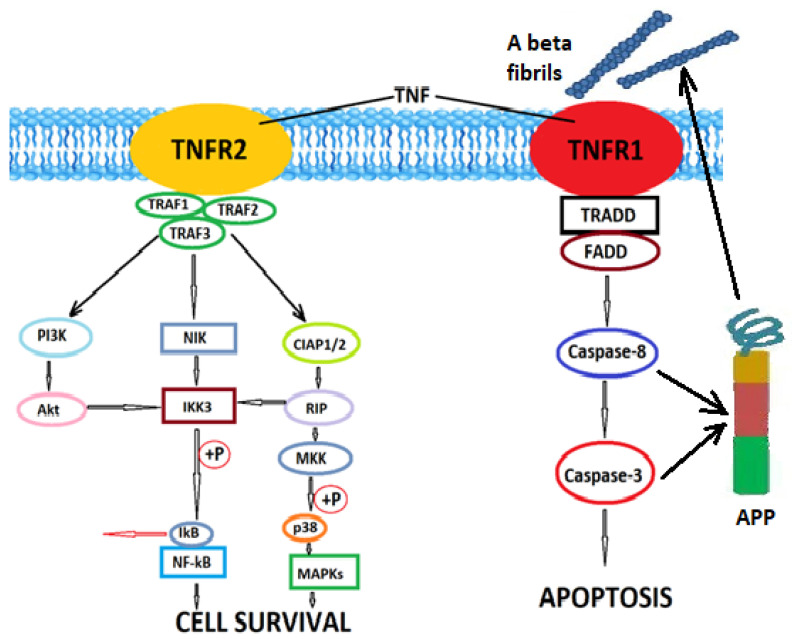
TNF signaling in Alzheimer’s disease. TNF receptor 1 (TNFR1) contains an intracellular TNF-receptor associated death domain (TRADD), which, upon TNF binding associates to FAS-associated death domain (FADD) and activates caspase 8 and caspase 3 leading to apoptosis. TNF receptor 2 (TNFR2) interacts with TNF receptor-associated factors (TRAF1, TRAF2, TRAF3) which interact with cellular inhibitor of apoptosis proteins 1 and 2 (CIAP1/2), NF-κB-inducing kinase (NIK) and phosphoinositide 3-kinase (PI3K), promoting cell survival. Akt—serine-threonine kinase; IκB—inhibitor of kappa B; IKK3—IκB kinase 3; NF-κB—nuclear factor kappa B; RIP—receptor interacting protein; MKK—mitogen-activated protein kinase; MAPKs—phosphorylated mitogen-activated kinases;. In AD, Aβ can physically interact with TNFR1 and promote neuronal death, while the caspases activated by TNFR1 signaling can cleave amyloid precursor protein (APP), increasing the Aβ load in a feed-forward loop.

Although still under research, TNF signaling is also impaired in AD. TNF-α binds to two receptors: a 55-kDa TNF receptor 1 (TNFR1), ubiquitously expressed in all cell types and which preferentially binds a soluble protein fragment of TNF, and a 75-kDa TNF receptor 2 (TNFR2), which is expressed predominantly in cells of the immune system and endothelial cells, and which is activated by the transmembrane form of TNF. TNFR1 contains an intracellular death domain and promotes inflammatory and pro-apoptotic signaling pathways, while TNFR2 interacts with TRAF2 (TNF receptor -associated factor 2) and modulates neuroprotective and regenerative pathways [146]. These pathways are schematically represented in Figure 2.

Convincing evidence has shown that TNF expression is increased and that TNFR1 levels are increased as well while TNFR2 levels decrease in AD [147]. In addition, TNF is more likely to bind to TNFR1 than to TNFR2 in AD, aggravating AD pathology [146].

## 4. Oxidative Stress and Neuroinflammation

Neuroinflammation is an innate response of the central nervous system (CNS) against harmful stimuli, mediated by the activation of glial cells, recruitment of peripheral leukocytes, and the production of proinflammatory secondary messengers (cytokines, chemokines, and ROS) [148]. It actually has two phases: a proinflammatory phase, aiming to neutralize the threat, and a late anti-inflammatory phase, that activates regenerative and healing processes [149]. However, chronic neuroinflammation has been shown in recent years to accompany and be involved in the pathogenesis of most neuroinflammatory diseases [150].

Research on the role of neuroinflammation in AD started with the identification of a rare coding variant in the *TREM2* (triggering receptor expressed on myeloid cells 2) gene that encoded an immunoreceptor tyrosine-based activation motif-containing cell surface receptor that increased the risk for developing AD four to five-fold [151]. Although the variant resulted in a single amino acid change in the extracellular domain of TREM2, the change hindered lipid ligand binding, leading to a partial loss of function [152]. Subsequently, large-scale genome-wide association studies identified a series of additional variants of microglia-expressed genes that influence the risk of AD, such as *SPI1, BIN1, INPP5D, ABCA7, SORL1, MS4A, CD2AP,* or *PICALM* [153].

Detailed studies in mouse models of AD described a subpopulation of transcriptionally distinct microglia, termed disease-associated microglia, which differed from the common homeostatic microglia [154] and supported the glial cells in overcoming the compromised bioenergetic state via mTOR signaling [155]. As such, microglial state can significantly impact the cerebral glucose utilization [156].

The chronic inflammatory state in the CNS is activated and maintained by several molecular pathways: the pattern recognition receptors (PRRs) pathway, cytokine receptor signaling, TREM2 signaling, and ROS-mediated pathway [157]. Mitochondria may be the link between the neurodegenerative and neuroinflammatory CNS pathology, participating in PRR signaling, ROS production, and inflammasome assembly [148,158].

### 4.1. PRR Signaling

PRRs are present on microglia, astrocytes, macrophages, and neutrophils, either as membrane receptors, such as the toll-like receptors (TLRs), or in the intracellular space, such as the nucleotide-binding oligomerization domain-like receptors (NLRs), or absent in melanoma 2 (AIM2)-like receptors [159]. They can bind pathogen-associated molecular patterns (PAMPs) released by microbial agents, or damage-associated molecular patterns (DAMPs) released by injured cells. Under stress conditions, the OMM may be damaged, leading to injuries of the mitochondrial IMM and release of mtDNA or cardiolipin into the cytoplasm [160]. Mitochondrial DNA is a molecule of double-stranded DNA which binds to cyclic GMP-AMP synthase, an enzyme which converts ATP and GTP into 2’3’-cyclic GMP-AMP. The latter molecule acts as a second messenger and activates stimulator of interferon genes (STING), leading to a conformational change of the protein which favors its phosphorylation by TANK-binding kinase 1 in the endoplasmic reticulum [161]. Further, STING phosphorylates IκB, the inhibitor protein of nuclear factor kappa-light-chain-enhancer of activated B cells (NF-κB), allowing the nuclear translocation of NF-κB and induction of inflammatory cytokines [148].

### 4.2. Inflammasome Assembly

The NLR (nucleotide-binding oligomerization domain and leucine-rich repeat-containing receptor) family receptors have a central nucleotide and oligomerization domain, a series of C-terminal leucine repeats, and N-terminal caspase (CARD) and pyrine (PYD) recruitment domains which mediate interactions for downstream signaling [162]. Following detection of dangerous molecules, the CARD domain binds to the adaptor apoptosis-associated speck-like protein containing CARD (ASC). Pro-caspase-1, contained in CARD, catalyzes to its active form and leads to the production of proinflammatory cytokines interleukin (IL)-1β and IL-18 and to cleavage of gasdermin D, thereby producing pores in the cell membrane and promoting pyroptotic cell death [163], which associates release of DAMPs [164]. NLRP3 (NLR family pyrin domain containing 3) inflammasome activation requires a priming signal inducing NF-κB transcriptional targets, followed by activating signals (mitochondrial dysfunction, ion dyshomeostasis, permeabilization of lysosomes by amyloid fibrils) [165]. Upon activation, the NLRP3 inflammasome relocates together with ASC from the cytoplasm to the mitochondria and MAMs. ROS, mtDNA or pathogenic protein aggregates, such as Aβ, can trigger NLRP3 activation, which in turn drives tau pathology [166] and enhances Aβ seeding via ASC specks released by pyroptosis, causing synaptic dysfunction [167].

### 4.3. Reactive Oxygen Species

ROS have important contributions in activating microglia, which in turn secrete proinflammatory cytokines and produce supplemental amounts of ROS in a vicious cascade. The released cytokines activate glial cells and stimulate ROS-induced apoptosis of pericytes, resulting in break-down of the blood brain barrier (BBB) [168].

Increased levels of Il-1β, which enhances the neuronal production of Aβ and induces phosphorylation of tau [169], were found in the brain, CSF, and serum of patients with AD [170]. IL-18 also enhances tau phosphorylation via increased expression of GSK3β and cyclin dependent kinase 5 [171].

The altered glucose metabolism is compensated in the brain by using fatty acids as alternative energy source, but the metabolism of these molecules further enhances IL-1β production, potentiating neuroinflammation [172].

### 4.4. The Role of TREM2 in Alzheimer’s Disease

Because recent research has pointed towards TREM2 as potential target in AD therapy, we shall briefly discuss the potential functions of the TREM2 receptor and its involvement in AD pathogenesis.

In the human genome, the gene encoding TREM2 is located within a cluster of genes at chromosome 6p21.1, together with TREM-like genes, namely TREML1 and TREML2 [173]. TREM2 and TREML2 appear to have opposing functions, as a missense variant of TREML2 protects against developing AD [174]. TREM2 is a transmembrane protein expressed in microglia and other immune cells that can be activated by lipids of the cell membrane, lipids from body fluids, or by components of lipoprotein complexes [152] including both lipidated and non-lipidated APOE, as well as nucleotides and negatively charged carbohydrates [175]. Upon ligand binding to TREM2, it associates to and dephosphorylates the signaling adaptor protein DAP12, followed by recruitment and activation of spleen tyrosine kinase (Syk) which activates PI3K causing elevation of intracellular calcium concentrations through Ca^2+^ release from the endoplasmic reticulum, and mitogen-activated protein kinases (MAPKs) [176]. The receptor has important contributions in promoting phagocytosis and clearing of pathogenic proteins and apoptotic cells, as TREM2 knock-out mice exhibited reduced clearance of Aβ_1-42_ aggregates and diminished the efficacy of antibody-mediated Aβ plaque clearing [177]. Indeed, microglial clustering around Aβ plaques has been described both in post-mortem AD brain tissue and animal models of AD with amyloid deposition. However, ablation of microglia in experimental setting did not affect the total amyloid burden [178], as opposed to genetic or pharmacological manipulation of the microglial activation state. IL-10 deficiency reduced amyloid plaques in APP/PS1 transgenic mice, while genetically-induced overexpression of IL-10 inhibited Aβ phagocytosis and led to increased amyloid burden [179]. It is still controversial whether TREM2 is important for resident microglia or in promoting infiltration of peripheral myeloid cells [180]. However, ultrastructural analysis of amyloid plaques with stochastic optical reconstruction microscopy described longer amyloid filaments in mice with reduced TREM2 expression [181], suggesting that microglia could compact amyloid fibrils, reducing neuronal process exposure to neurotoxic species of Aβ and limiting neuritic dystrophy [182].

TREM2 could also serve as a biomarker for AD. Currently, one of the earliest biomarkers of AD is a decrease of Aβ_42_ levels in the CSF, attributed to sequestration of Aβ_42_ in Aβ plaques [183]. However, although at risk for developing cognitive impairment, individuals with low Aβ_42_ levels in the CSF may be asymptomatic [184]. Proteolytic processing of TREM2 leads to the release of soluble fragments (sTREM2) that can be detected both in CSF and serum and which are increased in the CSF of AD patients, being correlated with levels of phosphorylated tau and tau pathology [185]. This finding indicates that significant microglial activation occurs after Aβ plaque deposition, leading to neuronal injury. As such, elevated sTREM2 levels may mark the transition from preclinical to symptomatic AD [173].

**Figure 3:**
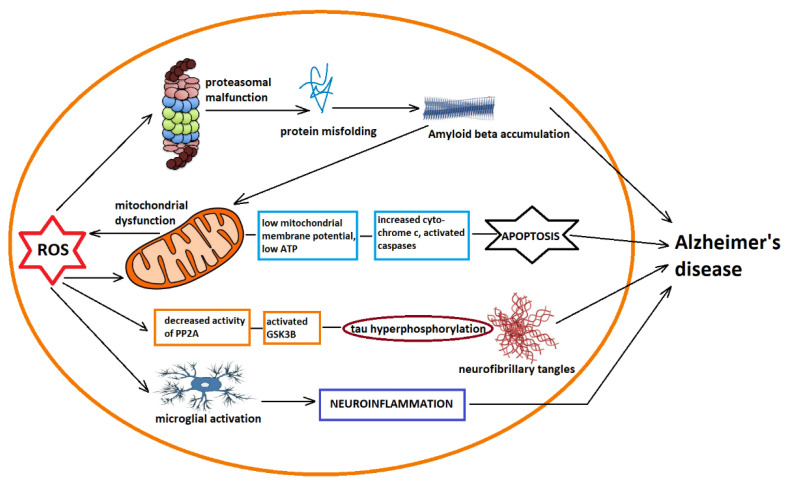
A complex relationship exists between oxidative stress which leads to mitochondrial dysfunction, that compromises mitochondrial bioenergetics and further increases oxidative stress. ROS also lead to protein misfolding and impair proteasomal function, causing accumulation of misfolded proteins and Aβ, which augments mitochondrial dysfunction. Through impaired enzymatic activity, various signaling pathways are altered, causing tau hyperphosphorylation. In addition, ROS activate microglia and neuroinflammation that further augments oxidative stress in AD.

Figure 3 Summarizes the complex relationships between oxidative stress, mitochondrial dysfunction and neuroinflammation discussed above.

## 5. Novel Therapeutic Strategies in Alzheimer’s Disease

Despite extensive research, the standard of care for AD patients still consists of symptomatic treatment, including cholinesterase inhibitors (donepezil, rivastigmine, and galantamine) and NMDA receptor blockers (memantine) [44]. None of these 4 drugs influences disease progression.

Decades of clinical trials driven by the amyloid cascade hypothesis and targeting Aβ yielded null results or even accelerated cognitive decline and caused toxic side effects [186]. These outcomes, together with a closer association of tau pathology with the regional and symptomatic progression of AD [8], shifted the attention toward tau-targeted therapies [187]. However, AD being a multifactorial condition, drugs with single mechanisms of action are unlikely to lead to favorable results in all stages of the disease compared to combinations of interventions that target different processes.

More detailed insight into the development and progression of AD revealed by research identified novel therapeutic targets and strategies, opening exciting perspectives. Studies in cell lines and animal models are encouraging, but much research is still needed before we can safely translate these findings into clinical practice. Some of these strategies targeting oxidative stress, mitochondrial dysfunction and neuroinflammation will be discussed in the following section.

### 5.1. Targeting Mitochondria and Mitochondrial Bioenergetics

The easiest way to improve mitochondrial biogenesis and dynamics is caloric restriction, which inhibits mTOR signaling via the AMPK-dependent pathway and activates SIRT1 [188]. Nonetheless, a series of exogenously delivered antioxidant molecules or compounds able to boost the endogenous antioxidant defense systems have been actively pursued in research.

Coenzyme Q10 is a cofactor of the ETC that helps maintaining ∆ψm, supports ATP synthesis and diminishes the amount of ROS generated by mitochondria. An analog of coenzyme Q10 is idebenone, which has a better pharmacological profile. Combining ubiquinone with a lipophilic cation moiety enhances the mitochondrial accumulation of ubiquinone. The molecule, MitoQ is water-soluble and can be administered orally. Tested in a mouse model of AD, it prevented cognitive decline and AD neuropathology [189]. However, a phase 1 clinical trial comparing coenzyme Q10 to a mixture of vitamin C, E, and alpha-lipoic acid versus placebo failed to show significant improvements (NCT00117403), while a planned study with MitoQ in mild cognitive impairment (NCT03514875) was withdrawn [190].

Vitamin E and MitoVitE protects mitochondrial membranes from oxidation by reducing H_2_O_2_ and prevents apoptosis by inhibiting cytochrome c release and caspase-3 activation [23]. However, in a randomized phase 3 clinical trial comparing the effect of memantine and vitamin E versus placebo in patents with AD (NCT00235716, TEAM-AD), there were no significant differences between the groups receiving memantine versus memantine and tocopherol [191]. Vitamin E also failed in preventing AD, as shown by an observational cohort study, PREADVISE (NCT00040378) [192].

Pramipexole acts as an antioxidant and accumulates in mitochondria scavenging free radicals [44]. To date only a phase 2 safety study has been performed, including 20 participants (NCT 01388478) [190].

A series of dietary phytochemicals, such as resveratrol, curcumin, or sulforaphane act by targeting multiple pathways. They exhibit anti-inflammatory properties, improve mitochondrial biogenesis and potentiate endogenous antioxidant pathways such as the Nrf2/ARE pathway. However, they have poor bioavailability [193], a drawback which could be overcome by engineering methods. Sulforaphane is currently tested in a randomized clinical trial (NCT04213391) estimated to be completed in December 2022 [190]. Resveratrol was tested in two completed studies (NCT00678431 and NCT01504854) and was shown to be safe and well tolerated but hardly able to influence cognitive performance [194] despite reducing markers of neuroinflammation [195]. Several clinical trials with curcumin are listed in the clinical trial registry [190], but the status of some of them is unknown. Both completed trials with published results (NCT00099710 and NCT00164749) were unable to demonstrate clinical efficacy [196].

Bezafibrate, a drug currently used to treat dyslipidemia, is also a PPAR agonist shown to improve mitochondrial biogenesis [197], which makes it a promising therapeutic agent in many neurodegenerative diseases, but which has not been tested yet in clinical setting.

Szeto-Schiller peptides (SS) are small tetrapeptides that penetrate to the IMM rather than targeting the mitochondrial matrix and can inhibit lipid peroxidation and ROS production [198]. In cell cultures and animal models of neurodegenerative diseases SS-31 (MTP-131, Elamipretide, Bendavia) upregulated mRNAs for ETC complexes I, IV, and V, for PGC-1α and TFAM, and improved mitochondrial dynamics by upregulating mitochondrial fusion proteins and downregulating proteins regulating fission.

In recent years, researchers have tried to improve mitochondrial dynamics by inhibiting excessive mitochondrial fission and improving fusion. Several Drp1 inhibitors, such as mitochondrial division inhibitor 1 (Mdivi-1), dynasore, P110, or diethyl (3,4 dihydroxyphenethylamino) (quinolyn-4-yl) methylphosphonate (DDQ) have been developed and studied in vitro and in mouse models [23]. Mdivi-1 increases mitochondrial biogenesis and reversibly inhibits complex I, decreasing ROS production [199], although its ability to inhibit mitochondrial fission in mammalian cells has been questioned [200]. Dynasore acts as a Drp1 inhibitor and a dynamin GTPase inhibitor [23,201]. It also regulates autophagy and decreases Aβ internalization and processing in the secretory pathway [23]. P110 blocks the interaction between Drp1 and Fis1, reduces ROS generation and restores the mitochondrial membrane potential [202], while DDQ decreases mitochondrial fission proteins and increases fusion proteins, also binding to Aβ and inhibiting its interaction with Drp1 [203].

Mitochondrial fusion can be enhanced with SAMβA (at least in cardiac cells) [204], BGP-15 (modulates OPA1 activity, at least in lung epithelial cells) [205], leflunomide (inhibits PARP and caspase-3 cleavage in mouse fibroblasts) [206], or M1, a molecule that proved neuroprotective in a Parkinson’s disease model [207]. However, much research regarding routes of administration, dosage, side effects, or stage of disease in which they might work is needed until any of these compounds will be ready to enter clinical testing.

### 5.2. Targeting Oxidative Stress and the Nrf2/ARE Pathway

As shown above, the Nrf2/ARE, the main cellular pathway regulating the antioxidant defense system, together with the TrkB pathway, which helps maintaining neuronal survival and synaptic plasticity, are downregulated in AD. Consequently, upregulating these pathways could help interrupt many of the vicious cascades involved in AD pathogenesis.

Melatonin, produced mainly by the pineal gland but also by lymphocytes, macrophages and monocytes, exhibits free-radical scavenging properties and is a potent inducer of the Nrf2 signaling pathway [208]. The levels of melatonin decrease in AD, with significant negative correlation with the Braak stages of the disease, which may explain the disrupted sleep-wake cycles of AD patients, which could indicate melatonin as a potential therapeutic strategy in early stages of AD [209].

A series of plant-derived molecules are able to neutralize ROS and strengthen the cellular defense systems, both phenolic compounds as well as non-phenolic ones. Although in preclinical studies these phytochemicals showed encouraging results, attempts to translate these findings to clinical studies may fail because most of them have poor bioavailability (due to chemical instability, rapid metabolism and clearance, poor ability to cross the blood brain barrier) [135]. Nanoparticle-mediated drug delivery may improve their pharmacokinetics and, along with appropriate drug dose regimens, could make them valuable tools in preventing and treating AD [193].

Because no clinical evidence of their effect in AD has been reported to date, we will provide a brief overview of the mechanism of action of these phytochemicals in Table 2.

### 5.3. Targeting TNFRs and Neuroinflammation

Existing therapies against TNF are monoclonal antibodies that have been approved in treatment in autoimmune and inflammatory diseases and that have been studied in more recent years in neurodegenerative diseases as well [146]. One such molecule is Infliximab, a chimeric IgG1 monoclonal antibody that binds to human TNF and that has been studied in mouse models of AD where it was delivered through intracerebroventricular injection. It was shown to reduce hyperphosphorylated tau, Aβ plaques, and TNF levels [222]. Another molecule is etanercept, a combination of the Fc portion of human IgG1 with the extracellular domain of TNFR2. Significant cognitive improvement was reported in an AD patient who also suffered of rheumatoid arthritis and was treated with etanercept [223]. Small, open-label studies with perispinal injections of etanercept also reported cognitive improvement in AD patients [224]. However, the single randomized, double-blind study, performed by Butchart et al. reported only non-significant differences between the treated arm and the control group at the expense of an increased risk for infections in the etanercept group [225]. Therefore, anti-TNF therapies are considered to be disadvantageous in neurological disorders.

Selectively regulating the two main types of TNF receptors could be more rewarding. Specific blockage of TNFR1 can be achieved with XPro-1595, a selective soluble TNF inhibitor [146]. The molecule has been studied in various animal models of autoimmune encephalomyelitis, multiple sclerosis, spinal cord injury, cerebral ischemia, Huntington’s disease, or Parkinson’s disease. Systemically administered XPro-1595 can cross the blood brain barrier and reach the cerebral tissue [226]. Used for AD, XPro-1595 reduced Aβ plaques and restored long-term potentiation in mice [227], also preventing synaptic loss if initiated in early stages of the disease [228]. TNFR1 signaling can be also inhibited with TNFR1-specific antibodies, such as ATROSAB [146]. It has not yet been tested for AD, but in a chemical lesion of the nucleus magnocellularis ATROSAB prevented microglial activation and shifted TNF signaling toward TNFR2, potentiating the neuroprotective pathways [229]. Another alternative would be to stimulate TNFR2 with specific agonists such as the soluble human TNFR2 agonist developed by Fischer et al. [230], which was shown to protect against oxidative stress-induced neuronal cell death. Another selective TNFR2 agonist, EHD2-scTNFR2, was tested in combination with ATROSAB in the nucleus basalis magnocellularis chemical lesion model by Dong et al., who showed that the combination strategy could be useful in treating acute neurodegenerative lesions caused by excitotoxicity [229].

Other molecules with anti-inflammatory actions have also been tested, such as masitinib or dasatinib, which are tyrosin kinase inhibitors. Following promising results of a phase 2 trial (NCT00976118) in mild-to-moderate AD, a phase 3 clinical trial was conducted on 721 participants (NCT01872598) between June 2013 and December 2020, but no results are posted or have been published [190]. However, a related molecule, dasatinib, is currently tested in a phase 2 trial in combination with the antioxidant quercetin.

Table 3 provides an overview of various drugs targeting neuroinflammation in clinical testing.

### 5.4. Cell-Based Therapies for AD

In view of the limited capacity for regeneration of the mammalian nervous system, cell-based therapy seems a promising strategy in various neurodegenerative disorders, AD included [231]. Researchers have used several stem cell types, such as embryonic stem cells (ESCs), adult or embryonic neural stem cells (NSCs), bone marrow derived stem cells (BMSCs), or mesenchymal stem cells (MSCs).

ESCs derive from the blastocyst’s inner cell mass and are pluripotent cells that can differentiate into various types of neurons. Unfortunately, they can lead to tumorigenesis [231] and the transplantation of NSCs isolated from embryos involves both ethical issues as well as safety problems related to the need for long-term culturing, possibility for differentiation into glial cells, purity of the NSC culture, dosage of cells and timing of transplantation [232]. By using retroviral transduction of four genes (two transcription factors—the octamer-binding transcription factor 4 and the sex-determining region Y-box, and two signaling factors regulating cellular proliferation and differentiation-the Kruppellike factor 4 and the avian myelocytomatosis viral oncogene homolog, or c-Myc), Takahashi and Yamanaka were able to generate induced pluripotent stem cells (iPSCs) from somatic cells [233] obtaining gene-matched cells which could be used in clinical applications [234]. By using specific proteins or inducers, these stem cells can subsequently differentiate into neural stem cells and ultimately into neurons [235]. However, these iPSCs retain a high proliferative capacity, may have chromosomal aberrations, and exhibit tumorigenic potential [231]. To overcome these issues, the pluripotent stem cell stage can be bypassed by transdifferentiation of proliferating somatic cells such as astrocytes into NSCs, either by genetically modifying the same four genes as for induction of iPSCs or by modulating a series of microRNAs (miRNAs) such as miRNA9 or miRNA-124 [236]. This process is faster, cheaper, and carries a lower risk for teratomas, but the heterogeneity of the resulting cells is a serious disadvantage [237].

MSCs can be derived from adipose tissue, tooth buds (from adult or embryonic sources), bone marrow, liver, or umbilical cord, cord blood and placenta [231,238]. Despite the greater propensity of bone marrow-derived stem cells to develop into osteocytes, in combination with galantamine nanoparticles they showed efficiency in a rat model of AD [239]. However, the clinical use of MSCs is restricted by the fact that donor age and long-term culture can negatively influence their differentiation and proliferation capacity [240].

Extracellular vesicles are membrane bilayer structures which carry proteins, lipids, miRNAs, and mRNAs that mediate the communication between cells and tissues [241] and regulate cell differentiation, immune response, angiogenesis, and tissue repair. The two main subtypes are exosomes and microvesicles. Exosomes form through inward budding of the plasma membrane, leading to the generation of endosomes that will subsequently fuse with the plasma membrane to secrete its content (DNA, lipids, RNAs, and cytosolic proteins) into the extracellular space [242]. Microvesicles are larger and directly bud from the plasma, followed by receptor–ligand interaction with neighboring cells and internalization by the recipient cell. They also contain mRNAs, miRNAs, cytosolic proteins and lipids [243]. In a mouse model of AD human umbilical cord mesenchymal stem cell-derived extracellular vesicles showed positive results by modulating microglial activation, reducing neuroinflammation and reducing amyloid deposition [244]. Extracellular vesicles can be modified by manipulating their parent cells, thereby incorporating specific miRNAs that can more potently target specific pathways impaired in AD [245].

Although much research is still needed and there is the potential for tumorigenesis through upregulating the Bcl-2 oncogene or activating the PI3K/Akt/mTOR or ERK/1/2 pathway [241], some cell-based therapies have already been approved and entered clinical trials. A completed phase 1 study using human umbilical cord-derived mesenchymal stem (NEUROSTEM) cells stereotactically injected into the hippocampus of AD patients showed the approach to be safe even on long-term follow-up visits (NCT01297218 and NCT01696591) [246]. Another completed phase 1/2 study showed safety and even efficacy of autologous adipose tissue-derived mesenchymal stem cells (AstroStem) delivered through IV infusion (NCT03117738) [232], while trial NCT02912169, evaluating IV and intranasal delivery of adipose-derived stromal cells in patients with AD, was withdrawn [193]. Trial NCT02054208 (a phase ½ trial) evaluated the safety and efficacy of intraventricular delivery of human umbilical cord blood-derived mesenchymal stem cells in low and high doses versus placebo, but no results have yet been posted, while the status of the follow-up trial (NCT03172117) is currently unknown [193]. The safety and efficacy of human umbilical cord-derived mesenchymal stem cells delivered IV was also the subject of trial NCT02672306, conducted in China, but whose status is currently unknown, as is the status of trial NCT02899091 conducted in South Korea, which also used mesenchymal stem cells. Table 4 shows the currently ongoing stem cell-based clinical trials.

### 5.5. Applications of nanotechnology in AD

Nanotechnology is an exciting research field with applications in AD both for early diagnosis and for drug delivery across the blood–brain barrier [231].

By using superparamagnetic iron oxide nanoparticles coated with the fluorescent curcumin or with antibodies against Aβ peptide as contrast agent in MRI imaging, Aβ plaques can be detected in vivo, and the sensitivity can be elevated by wrapping the nanoparticle in sialic acid [247]. These particles can be administered non-invasively, via intranasal route, bypassing the BBB. Magnetic core-plasmonic coat nanomaterials can be conjugated with Aβ antibodies and tau allowing for detection of both pathological proteins [248]. In addition, nanoparticles exposed to biological fluids are covered by a layer of proteins, known as the protein corona, which differs between healthy individuals and AD patients. Analysis of this protein corona can offer valuable information on disease stage and severity [249].

Nanostructures are also appealing drug delivery systems for AD, avoiding the need for increased doses and systemic toxic effects. They can be divided into organic nanostructures and inorganic ones.

#### 5.5.1. Organic Nanostructures

- Carbon nanotubes are cylindrical graphene sheets with a diameter of 1 nm and a length varying from 1 to 100 μm that have an impressive drug loading capacity. Depending on the arrangement of their graphene cylinders, they can be single-walled or multi-walled nanotubes [231]. Multi-walled carbon nanotubes have been successfully used to deliver berberine in a rat model of AD [250].
- Liposomes have low toxicity and are non-immunogenic, but are expensive, have poor stability and are rapidly removed by the reticuloendothelial system. To eliminate these drawbacks, solid nanoparticles and nanostructured lipid carriers were created in the 1990s. Phytochemicals such as quercetin [251], curcumin [252], or resveratrol delivered via solid nanoparticles showed increased and sustained levels of the drug in the brain with reduced formation of Aβ [252,253]. Nanostructured lipid carriers have also been successfully employed to deliver resveratrol [254] or curcumin for diminishing Aβ toxicity and improving the symptoms in AD models [255].
- Polymeric nanoparticles use highly biodegradable compounds, such as poly-ethylene glycol, poly-ethylenimine, poly-vinylpyrrolidone, poly-lactic acid, poly-lactic-co-glycolic acid or chitosan, which can modify their surface leading to improved drug delivery across the BBB. They have also been successfully used to deliver curcumin (leading to a six-fold increase of curcumin concentration in the brain compared to conventional delivery methods) [256] or donepezil, galantamine [257], rivastigmine [258], or memantine [259] to the brain, leading to improved efficacy and fewer side effects.

#### 5.5.2. Inorganic Nanoparticles

Inorganic nanoparticles are easy to synthesize, have controllable size, and low cytotoxicity, which has led to their increased use in recent years [232]. Among the metal-rich nanoparticles, gold nanoparticles have received considerable interest after research has shown that they can be used to dissolve Aβ aggregates by delivering thermal energy from a microwave field after the gold nanoparticles connected to Aβ [260]. Magnetic nanoparticles, iron–oxide (Fe_3_O_4_) nanoparticles coated with polyethylene glycol polyglycolide polymers, have the advantage of being targeted in the direction of an applied magnetic field and deliver the therapeutic molecule at specific sites, which makes them very attractive in treating malignant tumors [261]. Nonetheless, they can be applied in the treatment of neurodegenerative diseases as well. An iron oxide nanocomposite loaded with an anti-transferrin monoclonal antibody has been shown to dramatically decrease extracellular Aβ aggregation in vitro [262]. Quantum nanoparticles, or quantum dots, are nanosized semiconductor crystals containing cytotoxic cadmium salts, which limits their use mainly to diagnosis [232].

## 6. Conclusions and Future Directions

Over the past years, a series of candidate drugs for treating AD failed in clinical trials. Many of these targeted Aβ and neurofibrillary tangles. Various reasons for these failures have been identified and discussed.

One of these reasons would be that the therapeutic molecules targeted the wrong pathological substrates. The question whether the monomeric, oligomeric, or the fibrillary form of Aβ should be targeted is still open [263]. In the meantime, more detailed research on the vicious pathological cascades ignited in AD became available, involving mitochondrial dysfunction, oxidative stress, and neuroinflammation. Nonetheless, studies in cell lines or in rodent models, although cheap and expedite, may not exactly reproduce the development of AD pathology in human brains. Discrepancies need to be identified and bypassed.

Second, the intervention might be too late. AD pathology begins at least two decades before symptom onset [13]. As such, finding simple and preferably non-invasive tests to detect AD early would be the “Holy Grail” in AD treatment. Nanotechnology and other biomarkers, such as soluble TREM could be helpful in this direction. In fact, in 2018 the US Food and Drug Administration expanded the taxonomy of AD to contain four stages: stage 1—preclinical AD; stage 2—preclinical/prodromal AD; stage 3—prodromal AD; and stage 4—dementia [264]. It is likely that earlier interventions could increase the success rate.

Third, a more personalized approach, depending on the stage of the disease and the abnormally functioning pathways in each individual patient could be more rewarding since the “one size fits all” approach has failed. Specific biomarkers could identify these pathways, which could be normalized by using miRNAs and small interfering RNAs (siRNAs) delivered by exosomes to modulate these pathways [265].

Fourth, for improving delivery of therapeutic molecules across the BBB the recent advances in the field of nanotechnology could be efficiently used. As discussed above, phytochemicals are able to target multiple pathways, but their efficacy is hampered by the poor bioavailability.

Finally, genetic testing could identify the small percentage of carriers of genetic mutations known to cause early-onset AD. Using gene editing, these genes could be silenced, thereby delaying or even preventing the onset of the disease.

In conclusion, although much research is still needed regarding the therapeutic time window and stage of AD in which the various therapeutic strategies discussed above may have beneficial effects, the future holds promise. Enhancing mitochondrial biogenesis and dynamics may improve the overall energy metabolism of the CNS in very early stages of AD, delaying the ignition of the vicious cascades leading to AD pathology. Also in early stages, targeting neuroinflammation may promote phagocytosis of apoptotic cells and abnormal protein aggregates, preventing the propagation of neuroinflammation and loss of synapses while promoting cell survival. Improved delivery of antioxidants (with the use of nanotechnology) could reproduce the positive effects obtained in animal studies, especially if using molecules with multiple modes of action or cocktails of antioxidants. Stem cell therapy could aid in replacing lost neurons and glial cells and also promote endogenous neurogenesis, while genetically engineered extracellular vesicles and exosomes could deliver trophic factors and miRNAs to modulate specific pathways in different stages of AD.

## Figures and Tables

**Table 1:** Biomarkers of oxidative stress.

Substrate	Mechanism ROS and RNS-induced damage	Biomarkers
Proteins	1. O_2_^•−^ + NO^•^ ―> ONOO^−^ (peroxynitrite), which nitrates proteins mainly on tyrosine residues 2. Protein glycation by sugars and oxidation of amino acid side chains by ROS―> carbonyls	3-nitrotyrosine (3-NT) Protein carbonyls
Lipids	1. HO^•^, peroxy radicals (LOO^•^), alkoxy radicals (LO^•^), and alkyl radicals (L^•^) separate hydrogen atoms from fatty acid chains: L^•^ +O_2_―> LOO^•^LOO^•^ + LH ―> LOOH + L^•^; Subsequently the chain reaction propagates as long as labile H atoms are available	Lipid peroxides
	2. Lipid peroxides react with cell membrane proteins, generating aldehydes, 4-hydroxy-2-nonenal (4-HNE), 2-propene-1-al (acrolein) and malondialdehyde (MDA)	4-HNE, MDA
	3. lipid peroxidation of arachidonic acid (F2), eicosapentaenoic acid (F3) or docosahexaenoic acid (F4) generate isoprostanes	F2-, F3-, and F4-isoprostanes
DNA	ROS and RNS attack on guanine residues in DNA	8-hydroxy-deoxyguanosine (8OHdG)
RNA	ROS and RNS attack on guanine residues in RNA	8-hydroxyguanine (8OHG)

**Table 2:** Mechanism of action of phytochemicals with antioxidant effect.

Molecule(s)	Natural Source	Targeted Pathway	Outcomes	Reference
**Phenolic compounds**				
Sulfuretin	Flavonoid from the stem bark of *Albizzia julibrissin*	Nrf2/heme oxygenase-1; Pi3K/Akt	Decreased ROS, increased heme oxygenase 1; activated PI3K/Akt and Nrf2 pathway	[210]
Anthocyanins	Korean black beans	PI3K/Akt/nrf2 pathway	Increased phosphorylated PI3K, Akt, decreased hydrogen peroxide, 8-oxoguanine, cleaved caspase-3, inhibited PARP1, activated Nrf2 signaling	[211,212]
Resveratrol	Polyphenol from grapes and grapeseeds	PI3K/Akt/Nrf2 pathway	Diminished ROS and markers of lipid peroxidation, increased SOD, and GSH, activated PI3K, Akt, and heme oxygenase-1 and Nrf2	[213]
Tea polyphenols	Flavonoids from tea	TrkB/CREB/BDNF and KEAP-1/Nrf2 pathways	Decreased hydrogen peroxide, increased phosphorylated TrkB, BDNF, Phosphorylated Akt, SOD, GSH, catalase, activated Nrf2	[142]
Curcumin	*Curcuma longa* (turmeric)	PI3K/Akt and Nrf2 pathways	Scavenges ROS, increases SOD, catalase, GSH, decreases lipid peroxidation	[214,215]
Quercetin	Citrus fruits, apples, broccoli	Nrf2/ARE	Scavenges ROS, increases HO-1, SOD, catalase, thioredoxins	[216]
Naringenin and naringin	Citrus fruits, tomatoes, cherries	Nrf2/ARE pathway	Increased SOD, GSH, catalase	[217]
**Non-phenolic compounds**				
Acerogenin A	Stem bark of *Acer nikoense*	PI3K/Akt/Nrf2/HO-1 pathway	Diminished ROS, activated phosphorylated ASkt, Nrf2 and heme oxygenase-1	[218]
Brassica phenantrene	*Brassica rapa* ssp. campestris (turnip)	Nrf2-mediated expression of heme oxygenase-1 mediated by PI3K/Akt and JNK pathways	Increased HO-1, GSH, and nuclear translocation of Nrf2	[219]
Berberine	Roots, rhizome and stems of *Coptis chinensis,* barberry, goldenseal species	Nfr2/ARE pathway	Increased SOD and GSH, decreased ROS formation and markers of lipid peroxidation	[220]
Lycopene	Carotenoid in tomatoes, grapefruit	Nrf2/ARE pathway	Increased HO-1, SOD, catalase, GSH,	[221]

**Table 3:** Drugs in clinical testing phases targeting neuroinflammation [190].

Agent	Mechanism of Action	Trial Identifier	Sponsor
**Phase 1 trials**			
Edicotinib (JNJ-40346527)	Colony-stimulating factor 1 receptor antagonist, attenuates microglial proliferation	NCT04121208	Janssen, University of Oxford
Emtricitabine	Nucleoside reverse transcriptase inhibitor, reduces neuroinflammation	NCT04500847	Butler Hospital, Alzheimer’s Association, Brown University
Salsalate	Non-steroidal anti-inflammatory drug	NCT03277573	University of California, San Francisco
VT301	Targets regulatory T cells	NCT05016427	VTBIO Co.
XPro1595	TNF inhibitor	NCT03943264	Immune Bio, Alzheimer’s Association
**Phase 2 trials**			
AL002	Monoclonal antibody targeting TREM2Rs	NCT04592874	Alector, AbbVie
Baricitinib	Janus kinase inhibitor	NCT05189106	Massachusetts General Hospital
Canakinumab	Monoclonal antibody against IL-1β	NCT04795466	Novartis
Curcumin + aerobic yoga	Herbal extract with antioxidant and anti-inflammatory actions	NCT01811381	VA Office of Research and Development
Daratumumab	Monoclonal antibody targeting CD38 and regulating microglial activity	NCT04070378	Northwell Health, Janssen
Dasatinib + Quercetin	Tyrosine kinase inhibitor (dasatinib) and flavonoid with antioxidant action	NCT04063124	The University of Texas at San Antonio, Mayo Clinic
GB301	Targets regulatory T cells to reduce neuroinflammation	NCT03865017	Lifescience Australia
Lenalidomide	Reduces inflammatory cytokines	NCT04032626	Cleveland Clinic
Pepinemab (VX15)	Monoclonal antibody targeting semaphorin 4D	NCT04381468	Vaccinex, Alzheimer’s Association
Sargramostim	Granulocyte macrophage colony stimulating factor	NCT04902703	University of Colorado, Alzheimer’s association
TB006	Monoclonal antibody targeting galactin 3	NCT05074498	TrueBinding, Inc.
**Phase 3 trials**			
NE3107	MAPK inhibitor; reduces NFκB activation	NCT04669028	BioVie Inc.

**Table 4:** Stem cell therapies for Alzheimer’s disease in clinical trials [190].

Agent	Trial identifier	Phase	Sponsor
Allogenic human mesenchymal stem cells	NCT04040348	1	University of Miami
Autologous natural killer cells (SNK01)	NCT04678453	1	NKMax America
Human umbilical cord blood-derived mesenchymal stem cells (NEUROSTEM)	NCT03172117	1/2; extension phase	Medipost
CB-AC-02 (placenta derived mesenchymal stem cells)	NCT02899091	1/2	CHABiotech Co.
Allogenic adipose mesenchymal stem cell-derived exosomes	NCT04388982	1/2	Ruijin Hospital, Cellular Biomedicine Group
Allogenic human mesenchymal stem cells	NCT02833792	2	Stemedica

## Data Availability

Not applicable.
